# Optimizing Workplace Digital Mental Health Interventions: A Comprehensive Framework Based on Bayesian Meta-Analysis and Meta-Regression

**DOI:** 10.1192/j.eurpsy.2025.304

**Published:** 2025-08-26

**Authors:** N. Glozier, R. Morris, M. Deady, I. Choi, A. Milton, E. Stratton

**Affiliations:** 1 University of Sydney; 2 Black Dog Institute, Sydney, Australia

## Abstract

**Introduction:**

Digital mental health interventions have gained prominence as accessible and cost-effective solutions in workplace settings. However, our previous meta-analysis revealed a concerning trend: despite advancements in technology, the effectiveness of these interventions has not improved over time. This stagnation may be attributed to the significant and ongoing heterogeneity among interventions which indicates both variation in both sample and the intervention. We do not kow which theraputic approaches, design aspects or intervetnkion features enahnce efficacy.

**Objectives:**

We use a Bayesian meta-regression of an unpdated systematic review to develop a comprehensive framework to guide the design, development, and evaluation of workplace digital mental health interventions. By addressing the variability in intervention approaches and design and leveraging evidence-based practices, this framework seeks to enhance the quality and effectiveness of digital solutions for employee mental health.

**Methods:**

A systematic literature review was conducted to identify randomized controlled trials of employee based digital mental health interventions. Eligible studies were assessed based on specific criteria, including participant characteristics, intervention characteristics, and outcome measures. Data extraction and coding were performed, followed by a Bayesian meta-analysis approach. This method allowed for a more nuanced evaluation of the effectiveness of various intervention features and designs, accounting for uncertainty and prior knowledge in the field.

**Results:**

The review identified 95 interventions involving approximately ˜25,000 participants. The Bayesian meta-analysis confirmed small positive effects in reducing mental ill-health symptoms. Both sample and intervention characteristics contributed to heterogeneity across studies. Stress management and mindfulness interventions, particularly those designed with input from mental health experts, demonstrated more effiacy than CBT based approaches. Several intervention features, such as videos, feedback scores, and reminder texts, were associated with positive mental health outcomes.

**Conclusions:**

This review provides valuable insights into the optimal design and development of workplace digital mental health interventions. The identified framework and evidence-based practices offer guidance for developers to create effective interventions that address the heterogeneity within studies. Importantly, this framework has the potential to serve as a robust evidence base for app designers, enabling them to create more effective, personalised and engaging mental health applications.

**Disclosure of Interest:**

None Declared

